# Non-specific Health complaints and self-rated health in pre-adolescents; impact on primary health care use

**DOI:** 10.1038/s41598-020-60125-z

**Published:** 2020-02-24

**Authors:** Dorte Rytter, Charlotte Ulrikka Rask, Claus Høstrup Vestergaard, Anne-Marie Nybo Andersen, Bodil Hammer Bech

**Affiliations:** 1 Department of Public Health, Aarhus University, Bartholins Allé 2, 8000 Aarhus C, Denmark; 2 Department of Child and Adolescent Psychiatry, Research Unit, Psychiatry, Aarhus University Hospital, Palle Juul Jensens Boulevard 175, entrance K, 8200 Aarhus N, Denmark; 3 Department of Clinical Medicine, Aarhus University, 8000 Aarhus C, Denmark; 4 Research unit for general practice Department of Public Health, Aarhus University, Bartholins Allé 2, 8000 Aarhus C, Denmark; 5 Department of Public Health, University of Copenhagen, Øster Farimagsgade 5, 1014 Copenhagen K, Denmark

**Keywords:** Health care, Signs and symptoms

## Abstract

The objective of the present study was to explore past and future primary health care use in preadolescents reporting frequent non-specific health complaints or a low self-rated health compared to that of preadolescents with no frequent health complaints or with good self-rated health. The study was conducted as a cohort study based within the Danish National Birth Cohort (1996–2002). Information on non-specific health complaints and self-rated health was obtained by an 11-year follow-up questionnaire. Information about number of general practitioner (GP) contacts was obtained from the Health Insurance Service Register. A total of 44,877 pre-adolescents gave complete exposure information. Pre-adolescents who reported frequent non-specific health complaints had a higher use of GP compared to pre-adolescents without complaints across the five years following the index date (somatic complaints: IRR = (1.46 [1.38; 1.55], mental complaints: IRR = 1.16 [1.12; 1.19], both complaints: IRR = 1.58 [1.47; 1.69]). The same pattern was found for the association between low self-rated health and number of GP contacts (IRR = 1.41 (1.36; 1.46)). Non-specific health complaints and a poor self-rated health in pre-adolescents was associated with a higher past and future use of GP, indicating a need for development of early interventions with help for symptom management.

## Introduction

An increasing number of children and adolescents across nations in Europe and North America report non-specific health complaints such as headache, abdominal pain and back-pain^1–4^. In 2013–14, on average 27% of 11-year-old and 39% of 15-year-old European pre-adolescents and adolescents reported at least two non-specific health complaints on a weekly basis^3^. The prevalence varied widely between countries and was generally higher in girls.

The experience of frequent non-specific health complaints is an indicator of a low wellbeing. Hence, the symptoms may be viewed as different expressions of (bodily) distress, i.e. various responses to prolonged mental or physical stress in susceptible individuals^5^. For some children and adolescents, the non-specific health complaints can be very disabling and may affect every day functioning and result in excessive school absence^6^. In addition, the complaints have consistently been found to be associated with emotional problems such as anxiety and/or depression in cross sectional studies and to have implication for the future health of children with an increased risk of developing both somatic and psychiatric illness in adulthood^7–13^.

In both adults and adolescents, multiple non-specific health complaints have been shown to be associated with a lower self-rated health^14,15^. A low self-rated health in adults is predictive of higher health expenditures as well as a higher mortality rate compared to individuals reporting an excellent general health^16,17^. Self-rated health in adolescents has been found to be relatively stable through an 11-year period and a poor self-rated health in this age group has been shown to predict later health problems and use of medication in adulthood^18,19^.

Thus, preadolescents with frequent non-specific health complaints and poor self-rated health seem to constitute a growing and clinically relevant group of individuals who may acquire a maladaptive symptom-related behavior with increased use of health care and medication early in life^20^. Given that health behavior is established in childhood and adolescence, it seems relevant to investigate how self-rated health and the experience of frequent non-specific health complaints in youth affect the contact pattern to primary health care over time^21–24^. The few existing studies in this area have mainly been cross-sectional^7,25–29^.

The aim of the present study was to describe both past and future primary health care use in preadolescents reporting frequent non-specific health complaints or a low self-rated health compared to that of preadolescents who do not report these non-specific health complaints frequently or who rate their health as good.

## Method

### Study population and design

The study was based on longitudinal data on children from the Danish National Birth Cohort (DNBC)^30^. In short, women were recruited at their first pregnancy visit between 1996 and 2002. A total of 100,415 pregnancies were recruited resulting in 92,689 live born children after excluding live-born twins and triplets. In addition to multiple interviews during pregnancy and infancy, the children themselves and a parent were asked to fill in separate web-based questionnaires concerning health and lifestyle when the child turned 11 years. A total of 44,877 children gave complete information about non-specific health complaints and self-rated health at the 11-year follow-up.

Data from the 11-year follow-up was linked to information from the National Health Insurance Service Register (NHISR) using the unique personal identification number assigned to every citizen in Denmark^31^. The register holds information on services provided by health professionals in primary care contracted with the tax-funded public healthcare system. Since primary health care is free of charge in Denmark, private primary health care services not contracted by the tax-funded public health care system are considered negligible. Healthcare professionals must register all services provided with specific service codes to receive reimbursement for their services. From the NHISR it is possible to get information about type of contacts to the general practitioner (GP) (e.g. face-to-face consultation, e-mail and phone contacts) as well as the week and year of contact. The register also holds information on specific services provided by the GP, but no information on reason for the contacts.

### Measures

#### Exposure

Information about non-specific health complaints and self-rated health was obtained from the 11-year follow-up questionnaire. The presence of frequent non-specific health complaints was determined from questions concerning the frequency of experienced somatic and mental health complaints during the past 6 months. Somatic health complaints included headache, abdominal pain, dizziness and constipation, whereas mental health complaints included feeling sad, irritability or bad mood, nervousness and trouble falling asleep. The symptom checklist has previously been shown to have a high validity and reliability^32,33^. The children were asked to fill in how often they experienced the different health complaints (daily, more than once a week, almost every week, more than once a month, almost every month, “more seldom or never”). In this study, frequent experience of complaints was defined as experiencing at least one of the health complaints on a daily basis (yes/no)^4^. This was further categorized in four subgroups: (1) no health complaints, (2) only mental health complaints, (3) only somatic health complaints or (4) both somatic and mental health complaints. Furthermore, in order to investigate if the total burden of somatic and/or mental health complaints was associated with increased health care use, we also calculated a total mental and somatic complaints sum-score. I.e. for each symptom, a score depending on the frequency was given (6 for daily health complaints decreasing to 1 for more seldom or never) and finally summed for all somatic or mental complaints, respectively. Finally, the sum-scores were categorized into tertiles (for somatic sum scores: low ( = 4), medium (5–6), high (7–24) and for mental sum-scores: low (4–6), medium (7–10), high (11–24).

Self-rated health was defined according to the question: How would you rate your own health? The answer-categories excellent, good, reasonable and bad were dichotomized into good (excellent or good) and poor (reasonable or bad).

#### Outcome

The main outcome was defined as total number of “all contacts” to the GP per year, which was defined as the sum of face-to-face contacts, telephone contacts and home visits in both day-time and out of hours, and E-mail contacts. E-mail contacts were included in the NHISR from 2004. Contacts related to routine well-child visits and to the Danish childhood vaccination program were excluded.

In further sub-analyses, the contacts were separated into total daytime contacts (sum of home visits, face to face, telephone, and e-mail contacts), daytime face-to-face, telephone, and finally total out of hour contacts (sum of home visits, face to face and telephone contacts). In addition, specific commonly performed paraclinical tests (urine analysis, blood samples, C-reactive protein, Streptococcal antigen and spirometry) were included as separate outcomes.

#### Covariates

Covariates were all selected a-priori. Information about sex and date of birth was obtained from the civil registration system. Information about year of filling out the questionnaire, number of siblings (both full and half) and parental cohabitation was obtained from the 11 year follow-up questionnaire and information about maternal education and family income was obtained from Statistics Denmark (the year prior to the date for filling out the 11 year questionnaire). Somatic and mental morbidity in the child prior to filling out the questionnaire was identified from answers to the 11-year questionnaire to the parents as well as from the Danish National Patient Registry and the Danish Register of Medicinal Product Statistics from a predefined checklist (Supplementary methods).

#### Statistical analyses

Negative binomial regression analysis estimating incidence rate ratios (IRR) was used to investigate the association between self-reported frequent non-specific health complaints as well as poor self-rated health and both past and future primary health care use. The date for filling out the 11-year questionnaire was defined as the index date. IRRs were estimated for each year prior to the index date back until birth and for every year following the index date until 31/12/2014. Mean follow-up time after filling out the questionnaire was 1068 days. Cluster robust variance was applied to account for repeated measurements in the individual child. The IRRs were adjusted for sex, age at index date (10, 11, 12, 13 and 14 years), year at index date (2010, 2011, 2012, 2013 and 2014), number of full-siblings (0, 1, 2, 3+), number of half-siblings (0, 1, 2, 3+), parental cohabitation (yes, no), known somatic morbidity (yes, no), known mental morbidity (yes, no), maternal education (lower secondary, upper secondary, tertiary, unknown) and family income (in quartiles).

Sub-analyses were conducted to investigate the association between self-reported nonspecific health complaints and self-rated health respectively and type of GP contact (daytime contacts, out of hour contacts, face-to-face contacts, telephone contact and E-mail contacts). In addition, the association with specific services provided by the GP were investigated (urine stix, blood sampling, c-reactive protein, streptococcal antigen and spirometry). Finally, the main analyses were stratified on sex.

All statistical analyses were performed in Stata 13 (StataCorp LP, TX, USA).

#### Ethics

The study was approved by the Danish Data protection Agency under the Aarhus University comment agreement (j. number 2015-57-0002) and Aarhus University j. number 2016-051-000001, sequential number 528. According to the Committee on Health Research Ethics in the Central Denmark Region, no ethical approval was required for this study. The DNBC was approved by the Committee on Health Research Ethics in the Capital Region of Denmark j. number (KF) 01-471/94 and by the Danish Data protection Agency under the common agreement for Statens Serum Institut, j. number 2015-57-0102. Written informed consents to use the self-reported information with linkage to register information was obtained from all mothers on behalf of themselves and their child and all methods were carried out in accordance with relevant guidelines and regulations including the General Data Protection Regulation (GDPR).

## Results

### Description of study population

Children participating in the 11-year follow-up were similar to those lost to follow-up with regard to year of birth and primary health care use in their 11^th^ year of life. However, participants tended more often to be girls and to have mothers with longer educations and higher family income **(**Table 1**)**.

**Table 1:** Characteristics of participants and non-participants in the study based on the 11-year follow-up of the Danish National Birth Cohort.

	Non-participants (n (%))	Participants (n (%))	Total	p-value
n (row pct)	47812 (51·6)	44877 (48·4)	92689 (100)	
Gender (Girls)	21693 (45·4)	23499 (52·4)	45192 (48.8)	<0·005
Year of birth				<0·005
1996	59 (0·1)	78 (0·2)	137 (0.1)	
1997	332 (0·7)	397 (0·9)	729 (0.8)	
1998	6292 (13·2)	5307 (11·8)	11599 (12.5)	
1999	9114 (19·1)	10034 (22·4)	19148 (20.7)	
2000	10224 (21·4)	10335 (23·0)	20559 (22.2)	
2001	10041 (21·0)	9272 (20·7)	19313 (20.8)	
2002	9587 (20·1)	7756 (17·3)	17343 (18.7)	
2003	2163 (4·5)	1698 (3·8)	3861 (4.2)	
Mothers educational level				<0·005
Lower secondary/primary	5697 (11·9)	2501 (5·6)	8198 (8.8)	
Upper secondary	21097 (44·1)	16473 (36·7)	37570 (40.5)	
Tertiary	18835 (39·4)	25385 (56·6)	44220 (47.7)	
Unknown	2183 (4·6)	518 (1·2)	2701 (2.9)	
Gross family income				<0·005
1st quartile	14930 (31·2)	8243 (18·4)	23173 (25.0)	
2nd quartile	12178 (25·5)	10994 (24·5)	23172 (25.0)	
3rd quartile	10577 (22·1)	12595 (28·1)	23172 (25.0)	
4th quartile	10127 (21·2)	13045 (29·1)	23172 (25.0)	
Number of daytime contacts (at 11^th^ year)				<0·005
0	14382 (30·1)	12860 (28·7)	27242 (29.4)	
1–2	17370 (36·4)	16998 (37·9)	34368 (37·1)	
3–5	10638 (22·2)	10082 (22·5)	20720 (22·3)	
+6	5422 (11·3)	4937 (11·0)	10359 (11·2)	
Number of out of hours contacts (at 11th year)				<0·005
0	37055 (77·5)	35516 (79·1)	72571 (78·3)	
1–2	9566 (20·0)	8526 (19·0)	18092 (19·5)	
3–5	1079 (2·3)	775 (1·7)	1854 (2·0)	
+6	112 (0·2)	60 (0·1)	172 (0·2)	

The mean age of the children included was 11·4 years at index, 52% were girls, 10·7% reported experiencing at least one of the non-specific health complaints daily and 5·2% reported a poor self-rated health.

Children who reported frequent non-specific health complaints tended to have fewer full siblings and more half-siblings and more often to have non-cohabitating parents compared to children who did not report frequent complaints. In addition, they were more likely to suffer from concurrent somatic and mental conditions and to have a lower family income and a mother with a lower education. This was particularly evident for children who experienced both frequent mental and somatic complaints **(**Table 2**)**.

**Table 2:** Characteristics of participants dependent on the experience of self-reported frequent non-specific health complaint in pre-adolescents. Presented as n (%).

	No frequent complaints	Frequent mental complaints only	Frequent somatic complaints only	Frequent both somatic and mental complaints	p-value
n (row pct)	40062 (89·3)	3414 (7·6)	862 (1·9)	539 (1·2)	
Gender (Girls)	20472 (51·1)	2013 (59·0)	633 (73·4)	381 (70·7)	<0·005
Age at index					0·01
10	1997 (5·0)	190 (5·6)	37 (4·3)	20 (3·7)	
11	30765 (76·8)	2663 (78·0)	642 (74·5)	416 (77·2)	
12	6329 (15·8)	477 (14·0)	153 (17·7)	87 (16·1)	
13	860 (2·1)	74 (2·2)	25 (2·9)	11 (2·0)	
14	111 (0·3)	10 (0·3)	5 (0·6)	5 (0·9)	
Index year					0·02
2010	4370 (10·9)	364 (10·7)	82 (9·5)	58 (10·8)	
2011	18199 (45·4)	1451 (42·5)	390 (45·2)	235 (43·6)	
2012	8315 (20·8)	744 (21·8)	198 (23·0)	126 (23·4)	
2013	7040 (17·6)	650 (19·0)	145 (16·8)	83 (15·4)	
2014	2138 (5·3)	205 (6·0)	47 (5·5)	37 (6·9)	
Number of full siblings					<0·005
0	4822 (12·0)	468 (13·7)	124 (14·4)	109 (20·2)	
1	20959 (52·3)	1788 (52·4)	437 (50·7)	267 (49·5)	
2	11917 (29·7)	944 (27·7)	241 (28·0)	127 (23·6)	
3+	2364 (5·9)	214 (6·3)	60 (7·0)	36 (6·7)	
Number half siblings					<0·005
0	32857 (82·0)	2662 (78·0)	665 (77·1)	364 (67·5)	
1	3558 (8·9)	365 (10·7)	112 (13·0)	70 (13·0)	
2	2296 (5·7)	250 (7·3)	55 (6·4)	61 (11·3)	
3+	1351 (3·4)	137 (4·0)	30 (3·5)	44 (8·2)	
Cohabitating parents (yes)	31415 (78·7)	2534 (74·5)	605 (70·3)	358 (66·7)	<0·005
Somatic comorbidity (Yes)	5926 (14·8)	674 (19·7)	135 (15·7)	125 (23·2)	<0·005
Mental comorbidity (Yes)	1277 (3·2)	266 (7·8)	34 (3·9)	56 (10·4)	<0·005
Gross family income					<0·005
1st quartile	9792 (24·4)	963 (28·2)	273 (31·7)	192 (35·6)	
2nd quartile	9988 (24·9)	874 (25·6)	225 (26·1)	132 (24·5)	
3rd quartile	10133 (25·3)	785 (23·0)	185 (21·5)	116 (21·5)	
4th quartile	10149 (25·3)	792 (23·2)	179 (20·8)	99 (18·4)	
Mothers education level					<0·005
Lower secondary/primary	2173 (5·4)	204 (6·0)	77 (8·9)	47 (8·7)	
Upper secondary	14678 (36·6)	1208 (35·4)	363 (41·0)	232 (43·0)	
Tertiary	22740 (56·8)	1964 (57·5)	427 (49·5)	254 (47·1)	
unknown	471 (1·2)	38 (1·1)	5 (0·6)	6 (1·1)	

### Non-specific complaints and GP contacts

**Figure 1:**
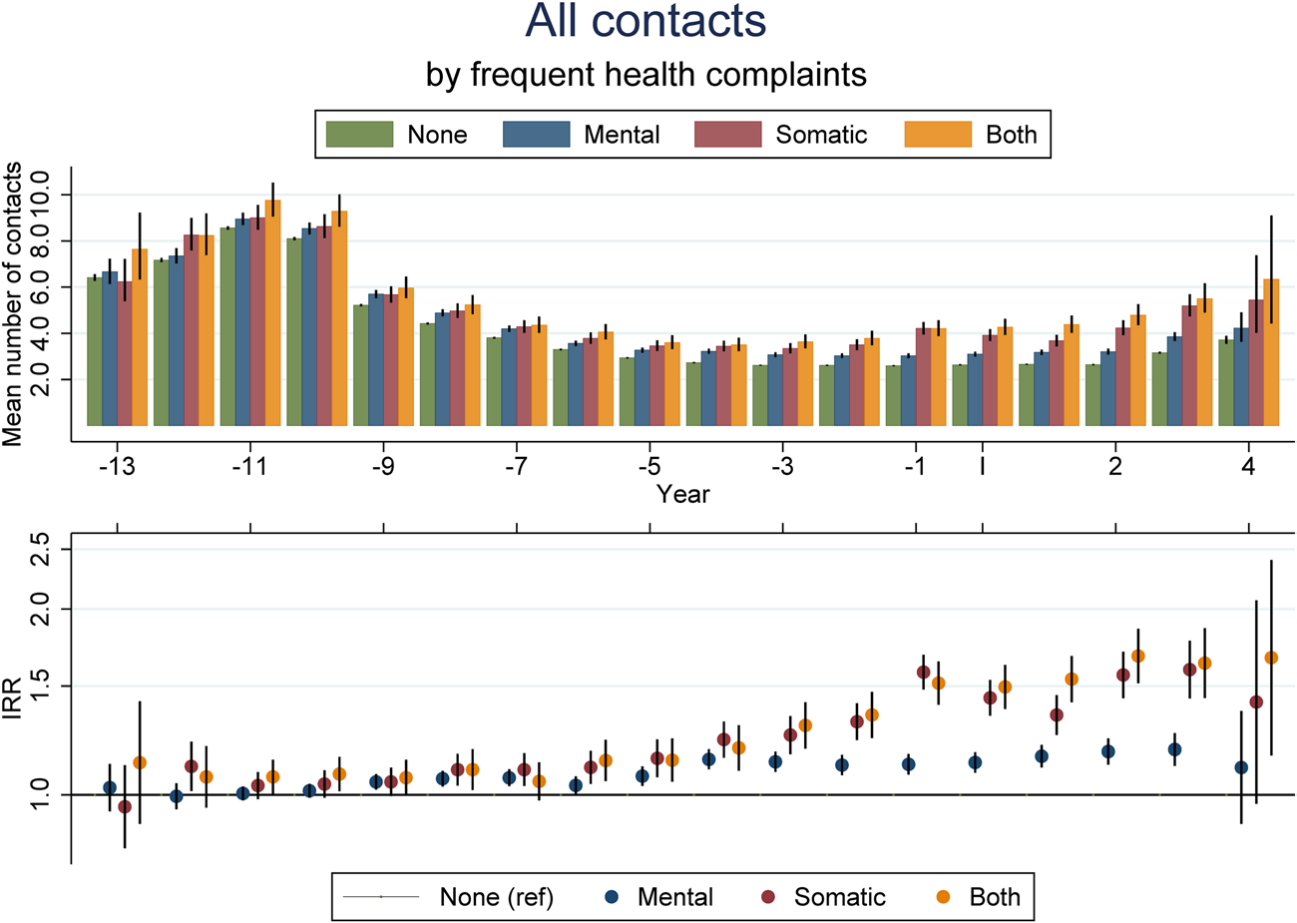
Mean number of yearly GP contacts by years before/since filling out the 11-year questionnaire according to the experience of frequent health complaints (non, somatic, mental or both somatic and mental) in top-panel and adjusted IRR (95%-CI) in bottom panel. IRRs and 95% CIs were estimated using negative binomial regression. Adjusted for sex, age, year, number of siblings, income and parental cohabitation.

Children suffering from daily non-specific complaints had more frequent contacts to the GP compared to children without daily complaints. This was particularly evident from 1 year prior to the index date and onwards, but the consultation rate ratio (IRR) was above 1 for several years prior to the index date (Fig. 1). The associations appeared strongest for pre-adolescents experiencing either daily somatic or both somatic and mental complaints. On average, these groups had 46% and 57% more GP contacts (IRR = 1·46 (1·38; 1·55) and 1·57 (1·47; 1·69, respectively) during the first 5 years following the index date. However, also pre-adolescents reporting frequent mental health complaints had a higher health care use compared to children who did not report frequent complaints (IRR = 1·16 (1·12; 1·19)). The associations were similar when split into the different types of GP contacts and for specific services provided (Supplementary efigure 1a–d and efigure 2a–e**)**. Also, the associations were similar for boys and girls (data not shown).

**Figure 2:**
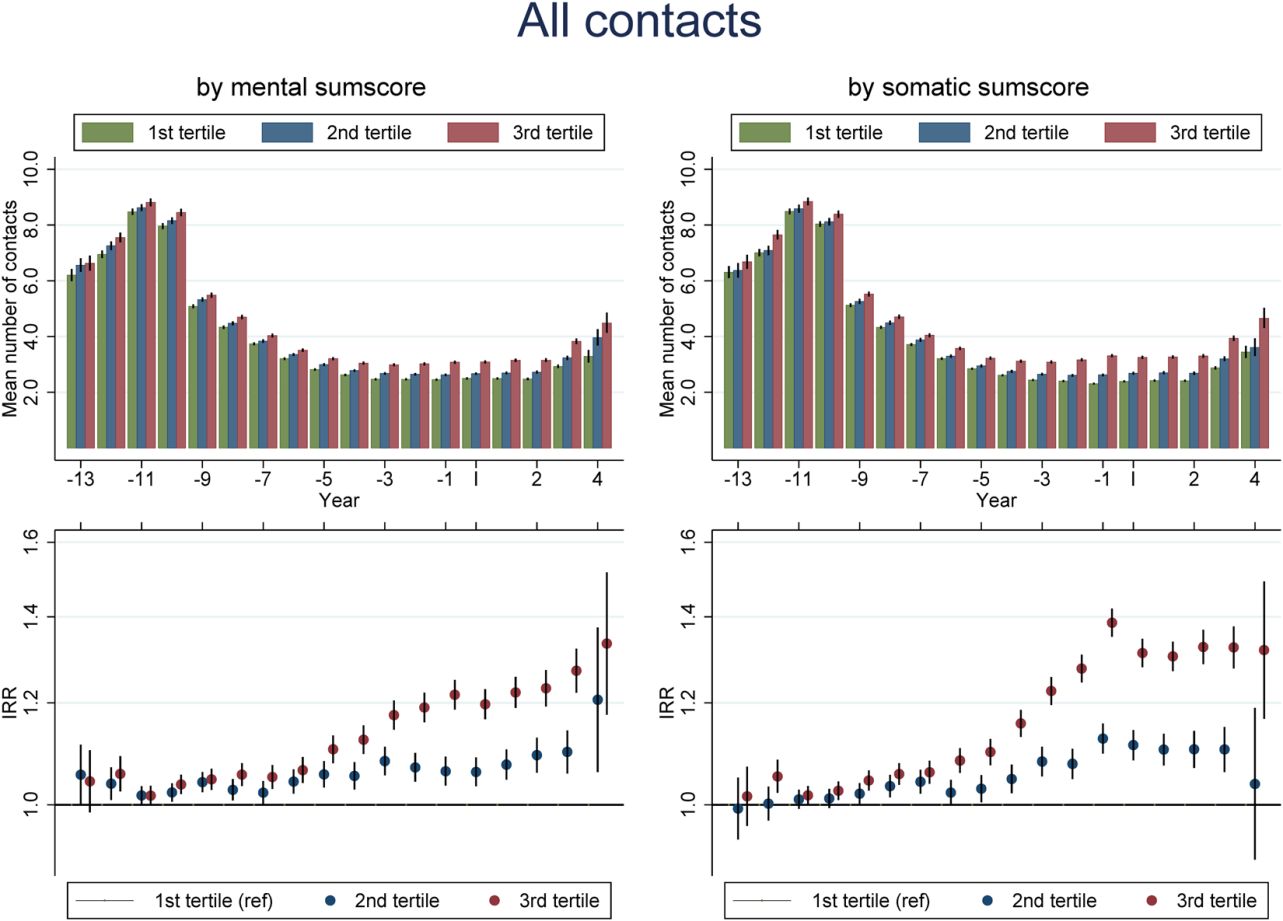
Mean number of yearly GP contacts by years before/since filling out the 11-year questionnaire according to burden of somatic (left panel) and mental (right panel) health complaints in top-panel and adjusted IRR (95%-CI) in bottom panel. The burden of somatic and mental health complaints was calculated as sum-scores depending on the frequency of symptoms reported and categorized into tertiles. IRRs and 95% CIs were estimated using negative binomial regression. Adjusted for sex, age, year, number of siblings, income and parental cohabitation.

The total burden of both non-specific somatic and mental complaints, i.e. the mental and somatic sum-scores, were also associated with primary health care use both prior to and following the index date, with an increase in IRR from early childhood and a leveling off around the index date (Fig. 2). Hence, children belonging to the second or third tertile for either the mental or somatic sum-scores had more GP contacts compared to children in the lowest tertile. The strongest associations were found for children in the third tertile.

### Self-rated health and GP contacts

**Figure 3:**
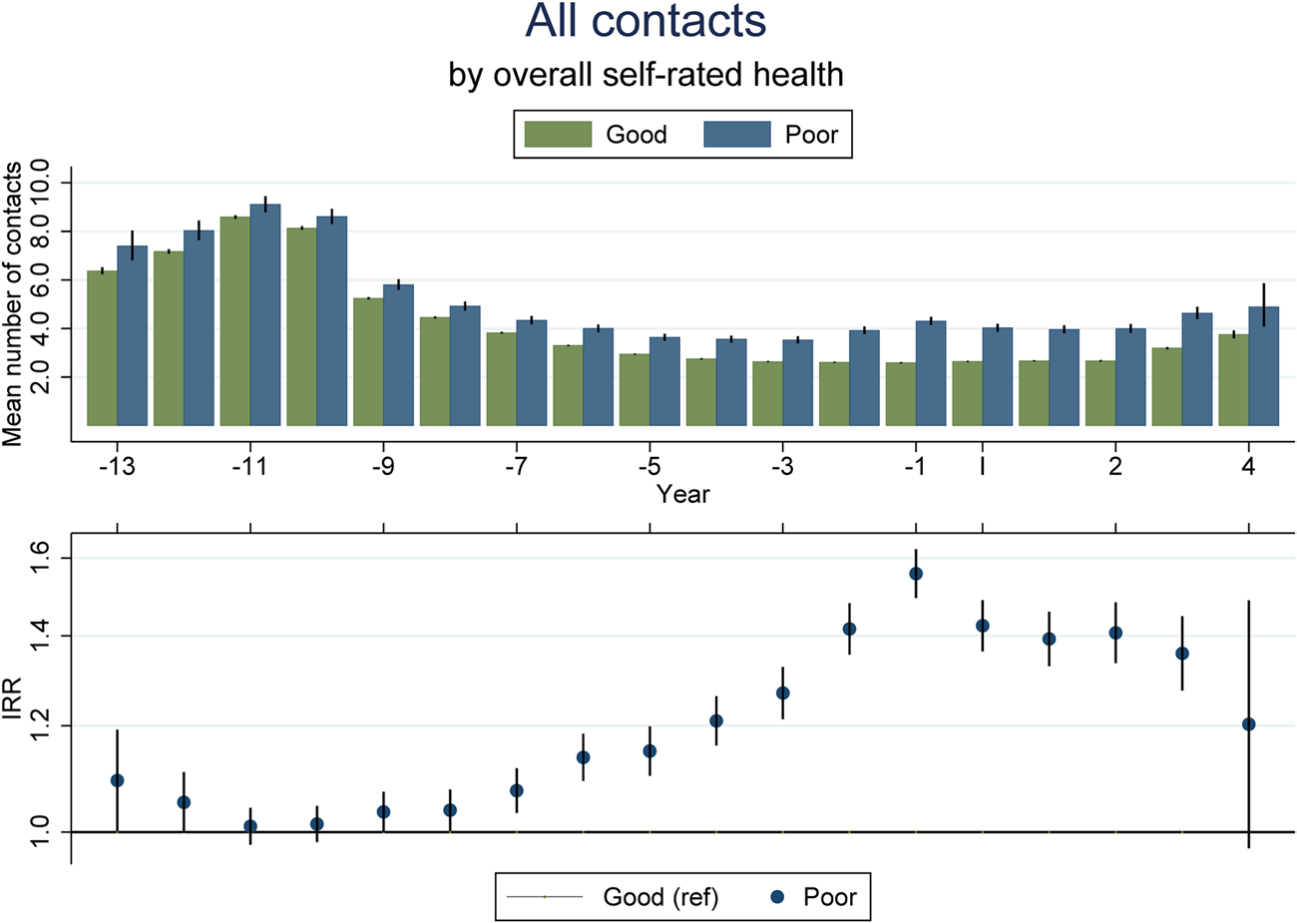
Mean number of yearly GP contacts by years before/since filling out the 11-year questionnaire according to self-rated health (good or bad) in top-panel and adjusted IRR (95%-CI) in bottom panel. IRRs and 95% CIs were estimated using negative binomial regression. Adjusted for sex, age, year, number of siblings, income and parental cohabitation.

Having a poor self-rated health was associated with a higher use of primary health care (Fig. 3). Again, the association was present several years prior to the index date and increased up until the index date before leveling off. Across the five years following the index date, pre-adolescents reporting a poor self-rated health had 41% more contacts on average compared to those who rated their health as good (IRR = 1·41 (1·36; 1·46)).The associations were similar when split into the different types of GP contacts and for specific services provided (supplementary efigure 3a–d and efigure 4a–e**)**. Also, the association was similar for boys and girls (data not shown).

## Discussion

In summary, the study found that children experiencing frequent non-specific health complaints in pre-adolescence had higher past and future primary health care use compared to children who did not experience frequent health complaints. The association appeared strongest for children with a high burden of non-specific health complaints. Likewise, children with poor self-rated health had a higher past and future health care use compared to children reporting a good self-rated health.

Most previous studies on the association between non-specific health complaints and health care use have been cross sectional^7,25–28^. They have consistently found that children reporting non-specific health complaints have a higher use of health care services. To our knowledge, only one previous study has investigated the association between non-specific health complaints and future health care use. This study found higher healthcare use in Danish children with functional somatic symptoms at the age of 5–7 years^29^. Functional somatic symptoms are comparable to non-specific health complaint, except that the former primarily refers to medically unexplained somatic complaints. In another study using the same cohort, health care use in early childhood was a predictor of consulting the GP with respect to functional somatic symptoms^34^. Together these findings are in accordance with results from the present study showing higher both past and future primary health care use in children reporting non-specific health complaints. This tendency was evident for all types of contact to the general practitioner and for most types of specific services provided, indicating that the reasons for contacts are heterogenic and of both urgent and non-urgent character.

To our knowledge, no prospective study has previously investigated the association between self-rated health in children and primary health care use. However, one study based in the Norwegian YOUNG-HUNT-study did report an association between self-reported health service attendance in adolescents and self-rated health 4 years later^15^. This is in line with the finding from the present study with more contacts to the GP many years prior to the index date in children with a poor self-rated health. Also, longer follow up of the YOUNG-HUNT study revealed that a poor self-rated health at the age of 16 was associated with both allostatic load 11 years later and redemption of prescribed medication 18 years later^18,19^. In summary, these studies indicate that a poor self-rated health is established in early life and that it remains a strong predictor of problematic health issues even into adulthood^21^.

Primary health care use in younger children is to a large degree predicted by maternal illness behavior^35,36^. This is the case for both health care use in general and for non-specific health complaints^24,37^. Since the young children rely on parents when seeking help, the higher use of primary health care services in children with non-specific health complaints in early childhood could be the result of a maladaptive illness behavior by the parents^10,21,22,38–40^. This may affect the child’s self-rated health and experience of non-specific health complaints later in life and this again may affect the child’s health care seeking^21^. Whether this is the explanation for the associations seen in this study or whether these children always have had objective health problems, is however not possible to conclude from the present study.

The strengths of the study include the large study population as well as the registry-based information about health care use over a long period of time. The registry-based information excludes the risk of recall bias and the long observation time gives a detailed picture of the temporal development in health care use. The findings are believed to be generalizable to countries with a similar primary health care service where the GP act as a gatekeeper, but reservation should be taken when generalizing to countries without tax-payed health care services.

There are however also limitations in the study. The attrition of potential study participants is relatively large (51·8%). Children who were lost to follow-up were more likely to be boys, to have mothers with a lower degree of education, to have lower household income and to have parents with mental illness. Also, although not directly comparable, the relatively large difference in the prevalence of daily non-specific health complaints between the present study and the Danish Health Behavior in School-aged children (HBSC) Study (approx. 12% vs 25%), could indicate that the children suffering from frequent non-specific health complaints were less likely to participate in the study. Participation however appeared to be independent of primary health care use in the 11^th^ year of life and selection bias is therefore expected to be limited. Another limitation of the study is that we do not know the reason for the health care contact. We also do not know whether the reported complaints and the poor self-rated health are due to known somatic or mental disorders. We did, however attempt to adjust for known concurrent morbidity according to both self-reported and registry-based information. This only changed the estimates marginally. Also, whereas the questions concerning non-specific health complaints have been validated among children in the relevant age-group, this is not the case for the question concerning self-rated health^32,33^. In adults, a poor self-rated health has been shown to be associated with an increased risk of becoming ill and die early and in adolescents it has been shown to be associated with health issues in adulthood. We therefore hypothesize that self-rated health also in 11-year-old children is an indicator of quality of life and future health.

In conclusion, children reporting frequent non-specific health complaints and poor self-rated health in pre-adolescence, have a higher both past and future health care use compared to children without such complaints and children who rate their health as good. With a general increase in the prevalence of non-specific health complains and poor self-rated health among children and adolescents, this is a group that needs special attention^4^. Development of early interventions are needed to prevent future morbidity and financial costs. These could be inspired by existing interventions, for medically unexplained symptoms in adults and should engage not only the young person but also the family as a whole^41–44^.

## Supplementary information

Supplementary Information.

## Data Availability

Due to restrictions related to Danish law and protecting patient privacy, the combined set of data as used in this study can only be made available through a trusted third party, Statistics Denmark. This state organisation holds the data used for this study. University-based Danish scientific organisations can be authorized to work with data within Statistics Denmark and such organisations can provide access to individual scientists inside and outside of Denmark. Requests for data may be sent to Statistics Denmark: http://www.dst.dk/en/OmDS/organisation/TelefonbogOrg.aspx?kontor=13&tlfbogsort=sektion or the Danish Data Protection Agency: https://www.datatilsynet.dk/english/the-danish-data-protection-agency/contact/.
