# Supplementary material for: Non-specific Health complaints and self-rated health in pre-adolescents; impact on primary health care use
Source: Sci Rep. 2020 Feb 24;10:3292. doi: 10.1038/s41598-020-60125-z (PMC7039989; doi:10.1038/s41598-020-60125-z)

Non-specific health complaints and self-rated health in pre-adolescents; impact on primary health care use

*Dorte Rytter[1], Charlotte Ulrikka Rask[2,3], Claus Høstrup Vestergaard[4], Anne-Marie Nybo Andersen[5], Bodil Hammer Bech[1]

Affiliations:

[1]Department of Public Health, Aarhus University, Bartholins Allé 2, 8000 Aarhus C, Denmark

[2]Department of Child and Adolescent Psychiatry, Research Unit, Psychiatry, Aarhus University Hospital, Palle Juul Jensens Boulevard 175, entrance K, 8200 Aarhus N, Denmark.

[3]Department of Clinical Medicine, Aarhus University, Denmark

[4]Research unit for general practice Department of Public Health, Aarhus University, Bartholins Allé 2, 8000 Aarhus C

[5]Department of Public Health, University of Copenhagen, Øster Farimagsgade 5, 1014 Copenhagen K.

Corresponding author:

Dorte Rytter, Department of Public Health, Aarhus University, Bartholins Allé 2, 8000 Aarhus C, Denmark.

Email: dr@ph.au.dk, Telephone number +4560381298.

Supplementary method: Morbidity in Children

Children were categorized as having somatic morbidity (yes/no) if they had any of the following conditions: asthma (parental report: “did your child ever have asthma”), heart disease (ICD10: DIxx), epilepsy (ICD10: G40), arthritis (ICD10: DM08), kidney disease (ICD10: DN0-39), diabetes (ICD10: DE10-14), intestinal disease (ICD10: DK50-51, DK900), serious vision or hearing disability (ICD10: DH54, DH90 and DH91) or diseases in the nerves, muscles etc.(ICD10: G80, DQ05, DG71)) and/or to have mental morbidity (yes/no) if they fulfilled the criteria of redemption of two prescriptions for any of the following psychopharmacological medications: ATC: N06A (anti-depressives), N05A (anti-psychotics), N06BA (ADHD), N05BA,N05BB, N03AX16 and N05BE01 (anxiety) with the exception of N06AA and N06AX12 and/or had one of the following psychiatric diagnoses (ICD10: DF20-DF50, DF80-89, DF90-98, DF99)) prior to filling out the questionnaire.

Figure legends for supplemental figures

efigure 1a: Mean number of yearly daytime GP contacts by years before/since filling out the 11-year questionnaire according to the experience of frequent health complaints (non, somatic, mental or both somatic and mental) in top-panel and adjusted IRR (95%-CI) in bottom panel. IRRs and 95% CIs were estimated using negative binomial regression. Adjusted for sex, age, year, number of siblings, income and parental cohabitation.

efigure 1b: Mean number of yearly out of hours GP contacts by years before/since filling out the 11-year questionnaire according to the experience of frequent health complaints (non, somatic, mental or both somatic and mental) in top-panel and adjusted IRR (95%-CI) in bottom panel. IRRs and 95% CIs were estimated using negative binomial regression. Adjusted for sex, age, year, number of siblings, income and parental cohabitation.

efigure 1c: Mean number of yearly daytime GP face to face consultations by years before/since filling out the 11-year questionnaire according to the experience of frequent health complaints (non, somatic, mental or both somatic and mental) in top-panel and adjusted IRR (95%-CI) in bottom panel. IRRs and 95% CIs were estimated using negative binomial regression. Adjusted for sex, age, year, number of siblings, income and parental cohabitation.

efigure 1d: Mean number of yearly daytime GP phone contacts by years before/since filling out the 11-year questionnaire according to the experience of frequent health complaints (non, somatic, mental or both somatic and mental) in top-panel and adjusted IRR (95%-CI) in bottom panel. IRRs and 95% CIs were estimated using negative binomial regression. Adjusted for sex, age, year, number of siblings, income and parental cohabitation.

efigure 2a: Mean number of yearly urine-stix examinations by years before/since filling out the 11-year questionnaire according to the experience of frequent health complaints (non, somatic, mental or both somatic and mental) in top-panel and adjusted IRR (95%-CI) in bottom panel. IRRs and 95% CIs were estimated using negative binomial regression. Adjusted for sex, age, year, number of siblings, income and parental cohabitation.

efigure 2b: Mean number of yearly blood samples taken by years before/since filling out the 11-year questionnaire according to the experience of frequent health complaints (non, somatic, mental or both somatic and mental) in top-panel and adjusted IRR (95%-CI) in bottom panel. IRRs and 95% CIs were estimated using negative binomial regression. Adjusted for sex, age, year, number of siblings, income and parental cohabitation.

efigure 2c: Mean number of yearly c-reactive protein examinations by years before/since filling out the 11-year questionnaire according to the experience of frequent health complaints (non, somatic, mental or both somatic and mental) in top-panel and adjusted IRR (95%-CI) in bottom panel. IRRs and 95% CIs were estimated using negative binomial regression. Adjusted for sex, age, year, number of siblings, income and parental cohabitation.

efigure 2d: Mean number of yearly streptococcal antigen examinations by years before/since filling out the 11-year questionnaire according to the experience of frequent health complaints (non, somatic, mental or both somatic and mental) in top-panel and adjusted IRR (95%-CI) in bottom panel. IRRs and 95% CIs were estimated using negative binomial regression. Adjusted for sex, age, year, number of siblings, income and parental cohabitation

efigure 2e: Mean number of yearly spirometry examination by years before/since filling out the 11-year questionnaire according to the experience of frequent health complaints (non, somatic, mental or both somatic and mental) in top-panel and adjusted IRR (95%-CI) in bottom panel. IRRs and 95% CIs were estimated using negative binomial regression. Adjusted for sex, age, year, number of siblings, income and parental cohabitation

efigure 3a: Mean number of yearly daytime GP contacts by years before/since filling out the 11-year questionnaire according to self-rated health in top-panel and adjusted IRR (95%-CI) in bottom panel. IRRs and 95% CIs were estimated using negative binomial regression. Adjusted for sex, age, year, number of siblings, income and parental cohabitation.

efigure 3b: Mean number of yearly out of hours GP contacts by years before/since filling out the 11-year questionnaire according self-rated health in top-panel and adjusted IRR (95%-CI) in bottom panel. IRRs and 95% CIs were estimated using negative binomial regression. Adjusted for sex, age, year, number of siblings, income and parental cohabitation.

efigure 3c: Mean number of yearly daytime GP face to face consultations by years before/since filling out the 11-year questionnaire according to self-rated health in top-panel and adjusted IRR (95%-CI) in bottom panel. IRRs and 95% CIs were estimated using negative binomial regression. Adjusted for sex, age, year, number of siblings, income and parental cohabitation.

efigure 3d: Mean number of yearly daytime GP phone contacts by years before/since filling out the 11-year questionnaire according to self-rated health in top-panel and adjusted IRR (95%-CI) in bottom panel. IRRs and 95% CIs were estimated using negative binomial regression. Adjusted for sex, age, year, number of siblings, income and parental cohabitation.

efigure 4a: Mean number of yearly urine-stix examinations by years before/since filling out the 11-year questionnaire according to self-rated health in top-panel and adjusted IRR (95%-CI) in bottom panel. IRRs and 95% CIs were estimated using negative binomial regression. Adjusted for sex, age, year, number of siblings, income and parental cohabitation.

efigure 4b: Mean number of yearly blood samples taken by years before/since filling out the 11-year questionnaire according to self-rated health in top-panel and adjusted IRR (95%-CI) in bottom panel. IRRs and 95% CIs were estimated using negative binomial regression. Adjusted for sex, age, year, number of siblings, income and parental cohabitation.

efigure 4c: Mean number of yearly c-reactive protein examinations by years before/since filling out the 11-year questionnaire according to self-rated health in top-panel and adjusted IRR (95%-CI) in bottom panel. IRRs and 95% CIs were estimated using negative binomial regression. Adjusted for sex, age, year, number of siblings, income and parental cohabitation.

efigure 4d: Mean number of yearly streptococcal antigen examinations by years before/since filling out the 11-year questionnaire according to self-rated health in top-panel and adjusted IRR (95%-CI) in bottom panel. IRRs and 95% CIs were estimated using negative binomial regression. Adjusted for sex, age, year, number of siblings, income and parental cohabitation

efigure 4e: Mean number of yearly spirometry examination by years before/since filling out the 11-year questionnaire according to self-rated health in top-panel and adjusted IRR (95%-CI) in bottom panel. IRRs and 95% CIs were estimated using negative binomial regression. Adjusted for sex, age, year, number of siblings, income and parental cohabitation

efig 1a. All contacts (daytime)

by frequent health complaints

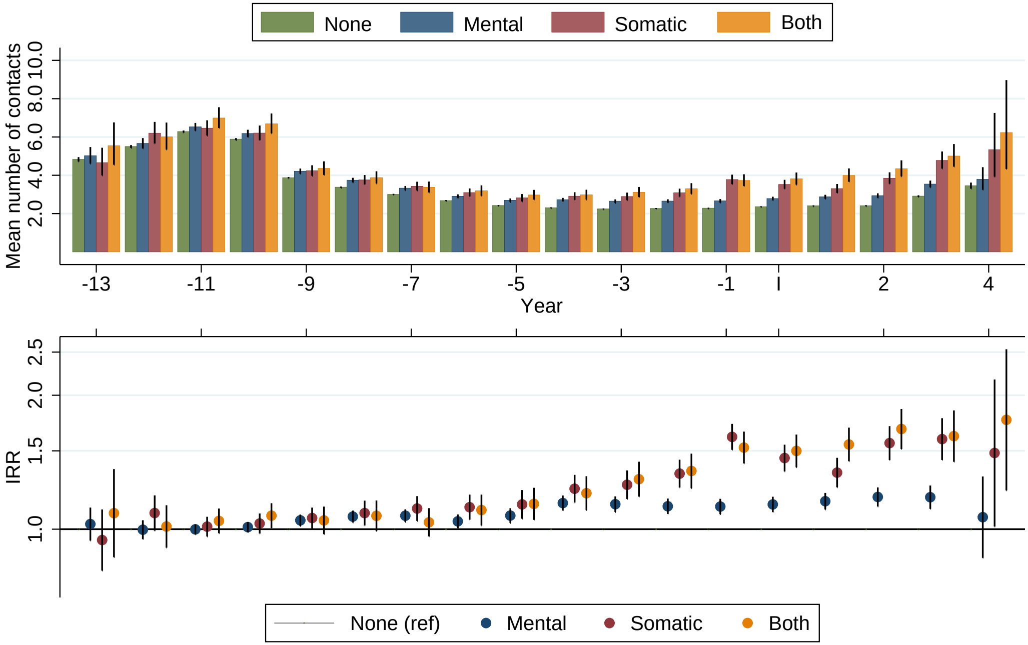

efig 1b. All contacts (OOH)

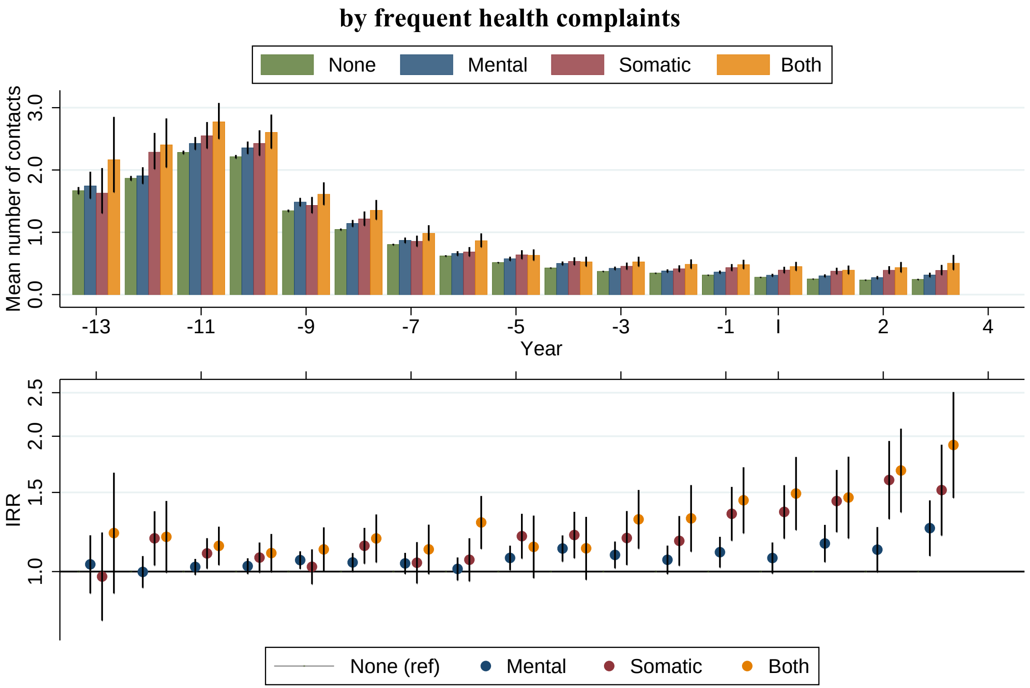

efig 1c. Face-to-face consultations (daytime)

by frequent health complaints

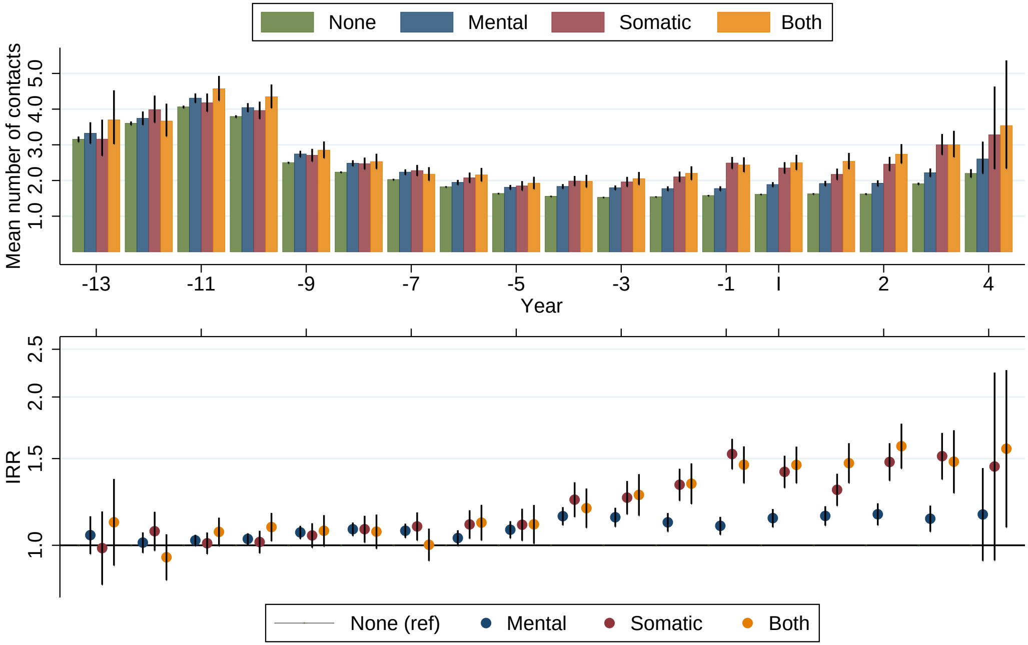

efig 1d. Daytime phone

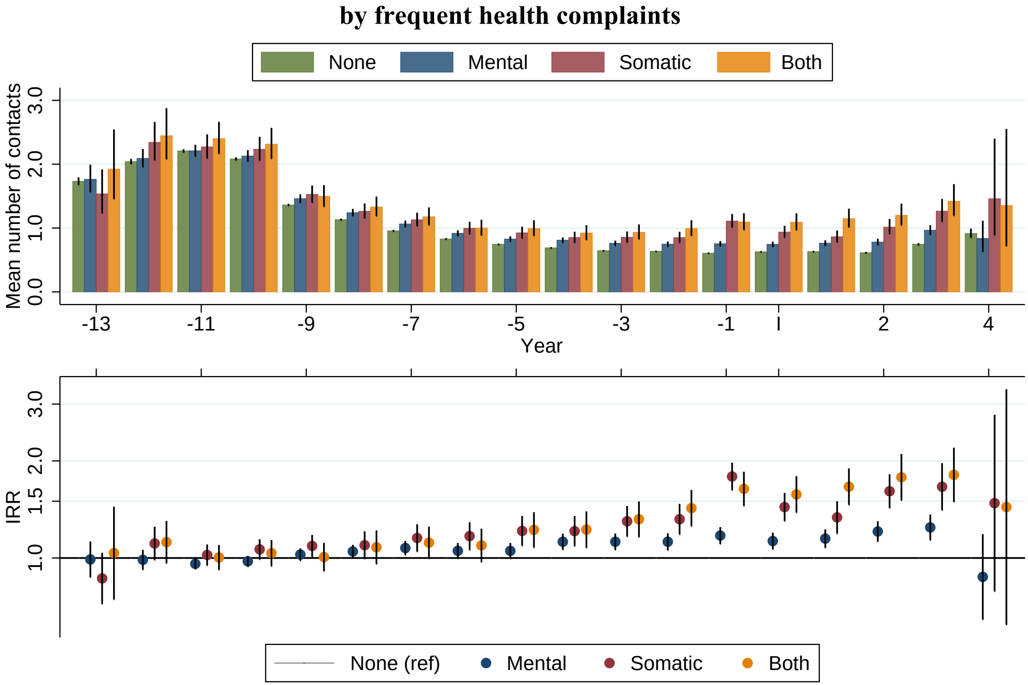

efig 2a. Urine analysis

by frequent health complaints

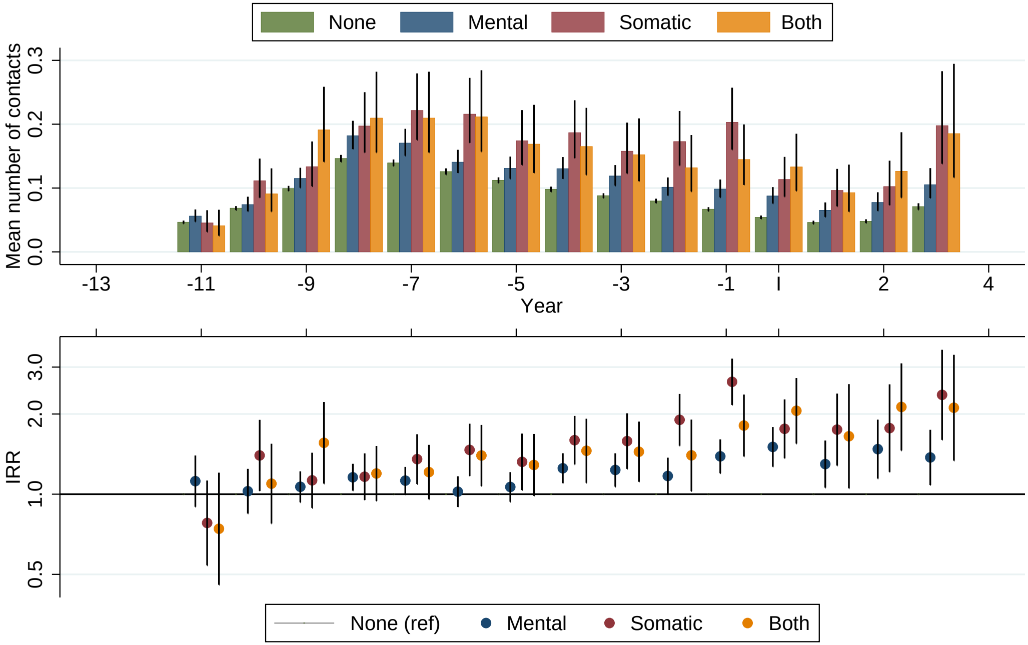

efig 2b. Bloodsamples

by frequent health complaints

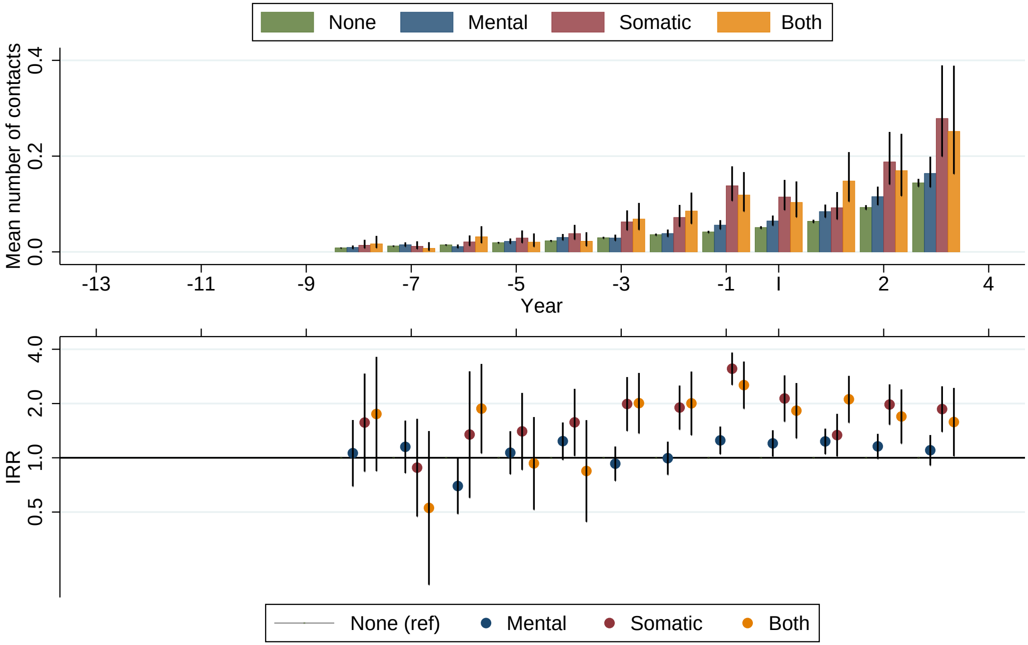

efig 2c. C-reactive protein

by frequent health complaints

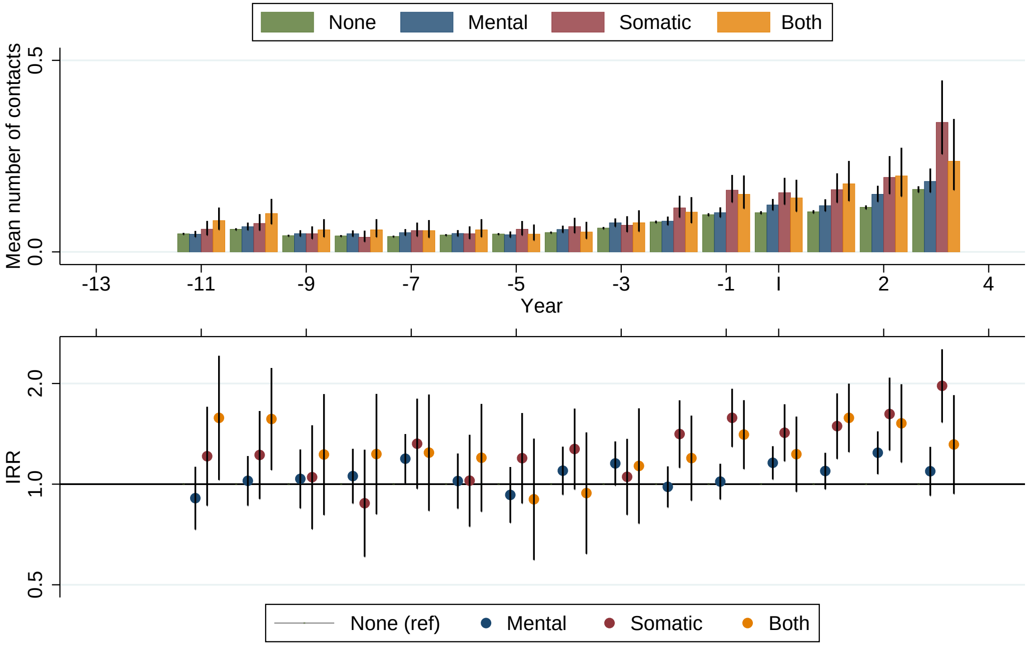

efig 2d. Streptococcal antigen

by frequent health complaints

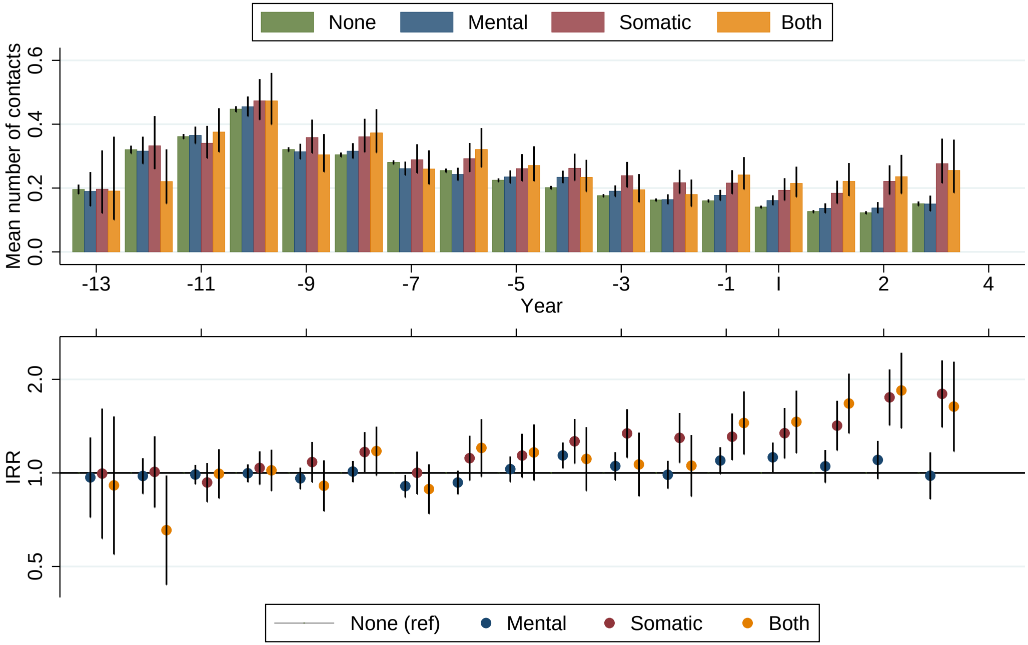

efig 2e. Spirometry

by frequent health complaints

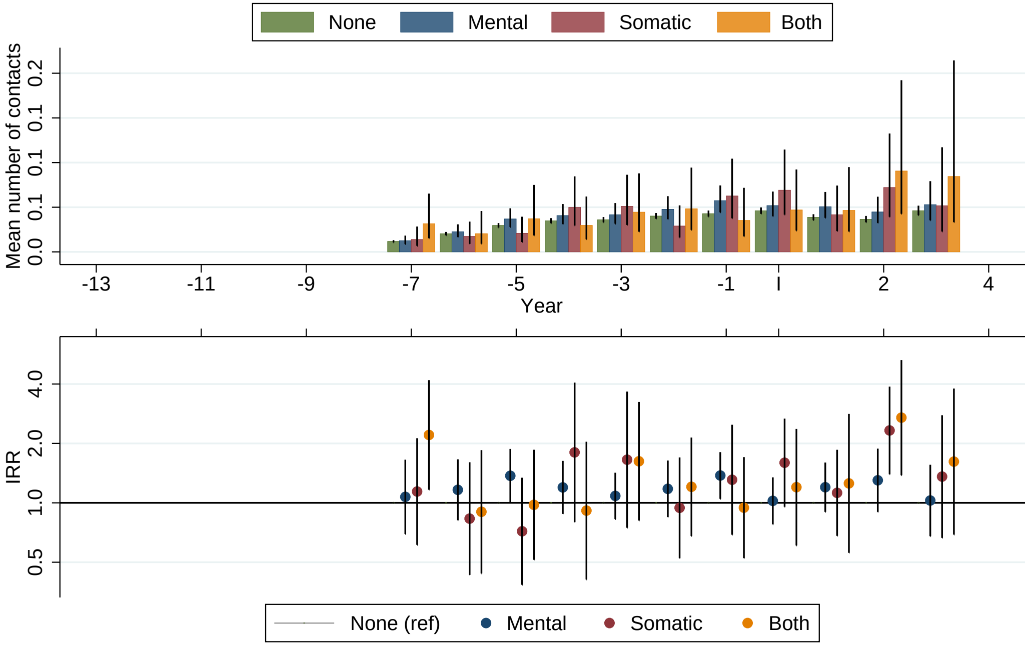

efig 3a. All contacts (daytime)

by overall self-rated health

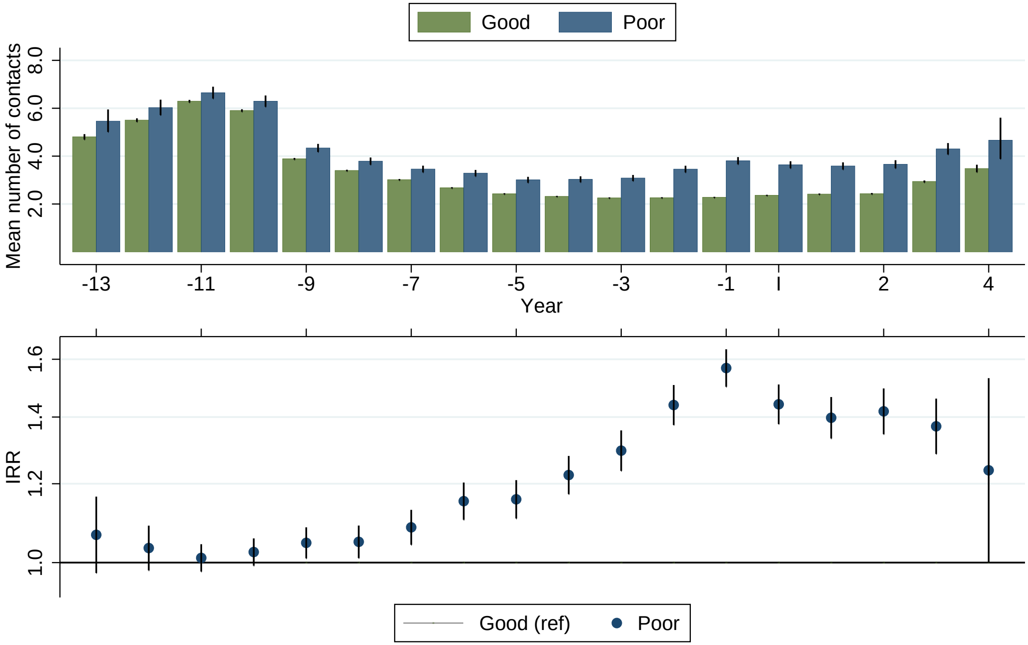

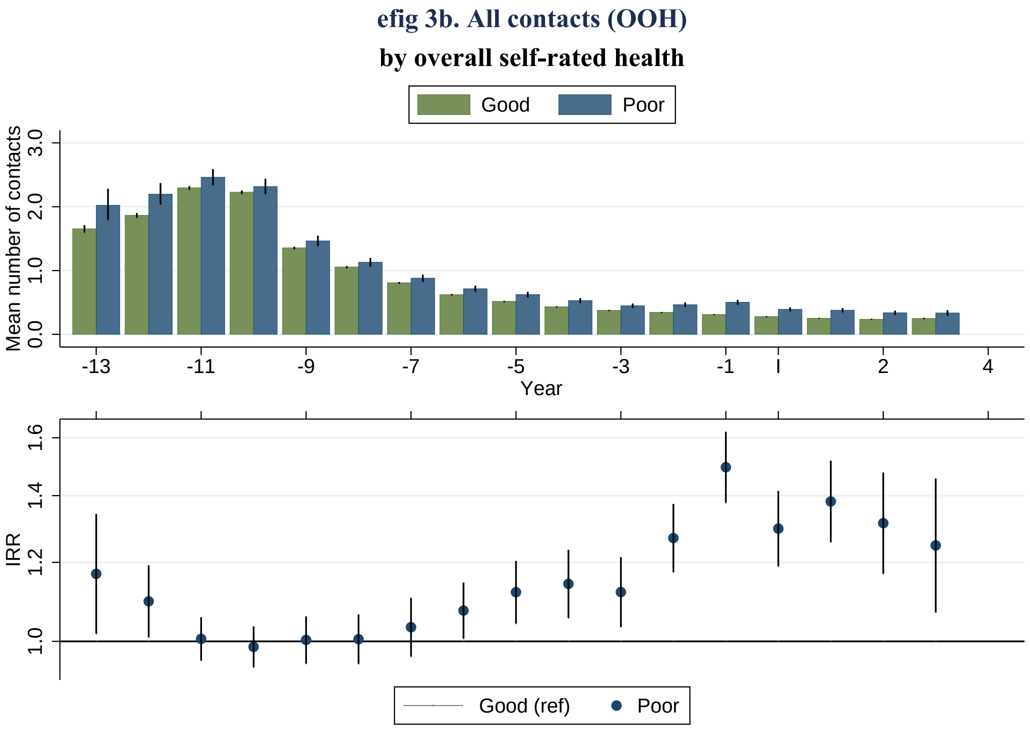

efig 3b. All contacts (OOH)
by overall self-rated health
Good
Poor
Mean number of contacts
0.0
1.0
2.0
3.0
-13
-11
-9
-7
-5
-3
-1
I
2
4
Year
IRR
1.0
1.2
1.4
1.6
Good (ref)
Poor

efig 3c. Face-to-face consultations (daytime)

by overall self-rated health

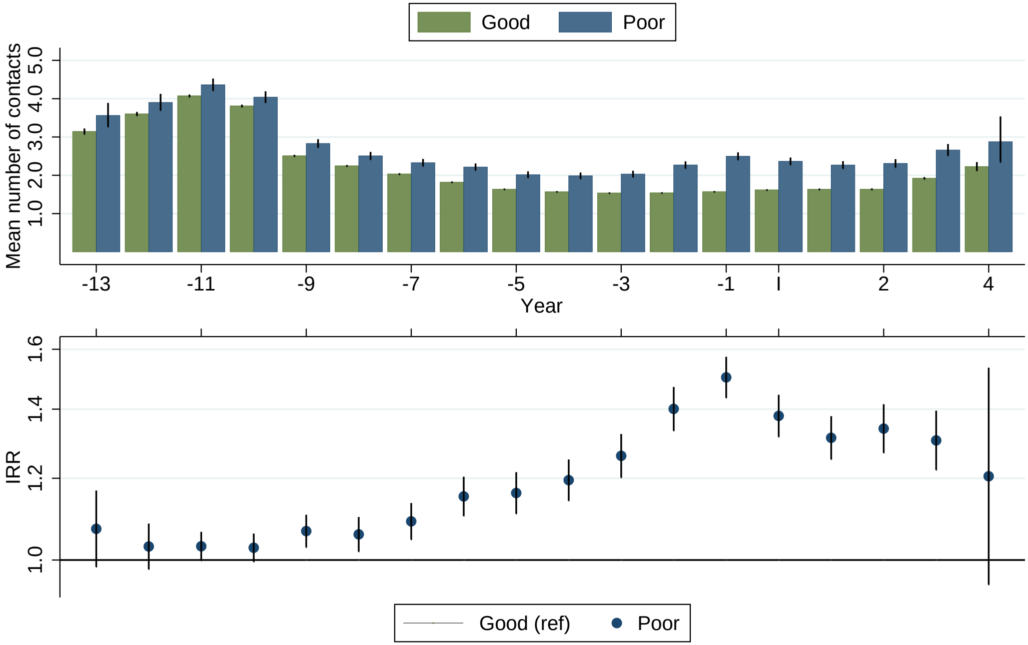

efig 3d. Daytime phone

by overall self-rated health

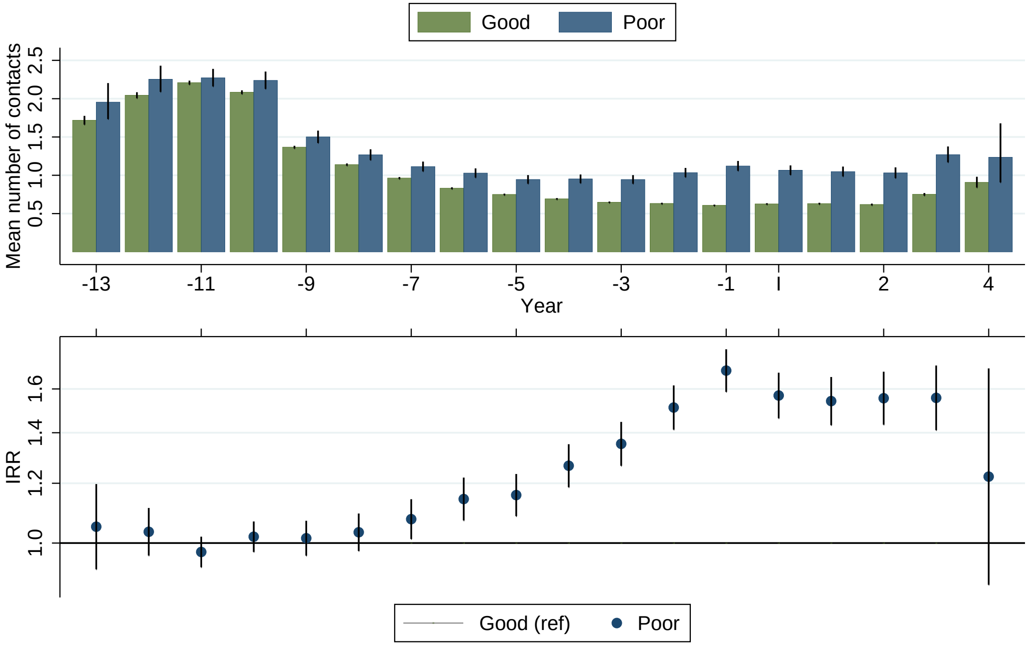

efig 4a. Urin analysis

by overall self-rated health

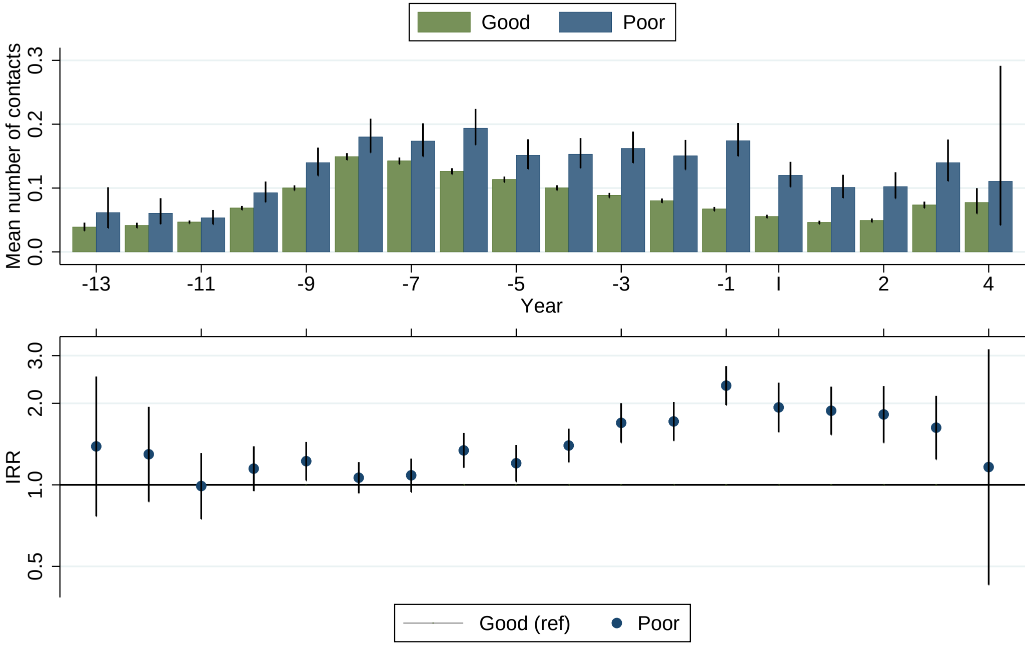

efig 4b. Bloodsamples

by overall self-rated health

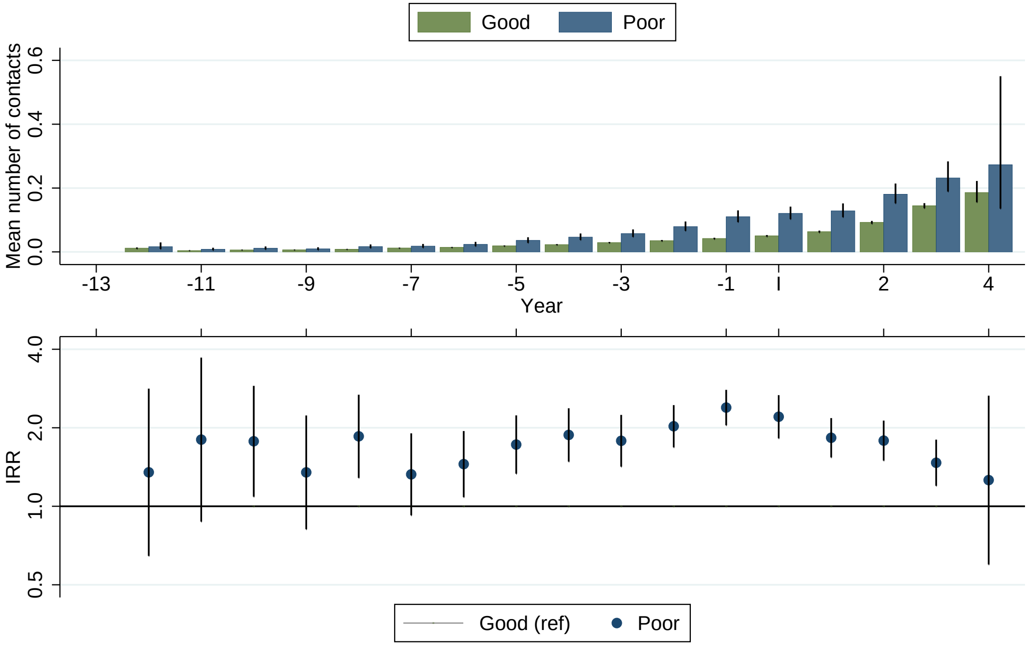

efig 4c. C-reactive protein
by overall self-rated health

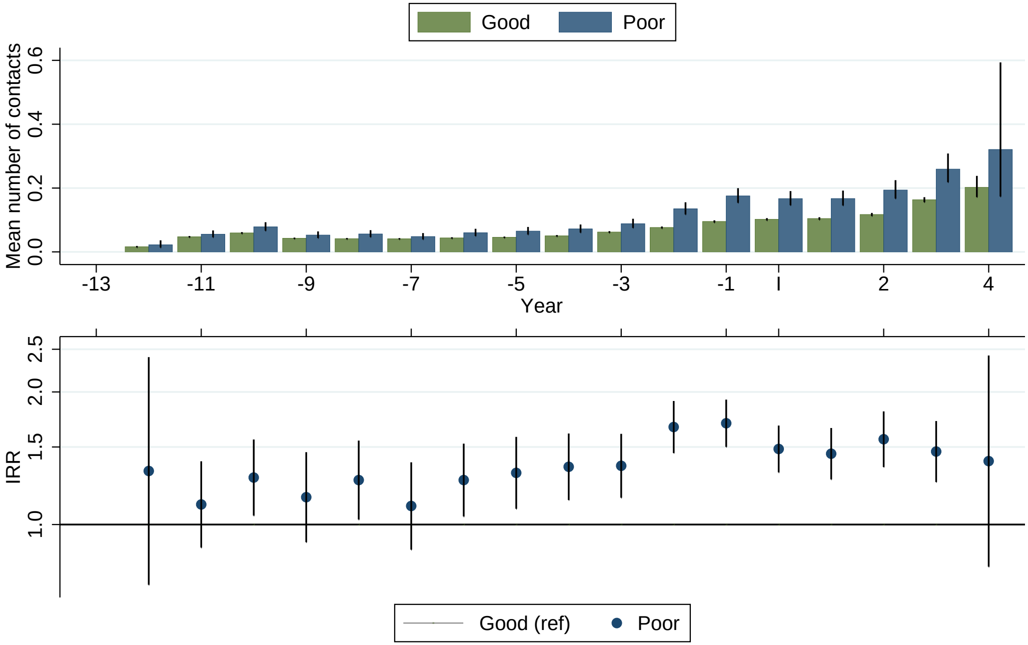

efig 4d. Streptococcal antigen

by overall self-rated health

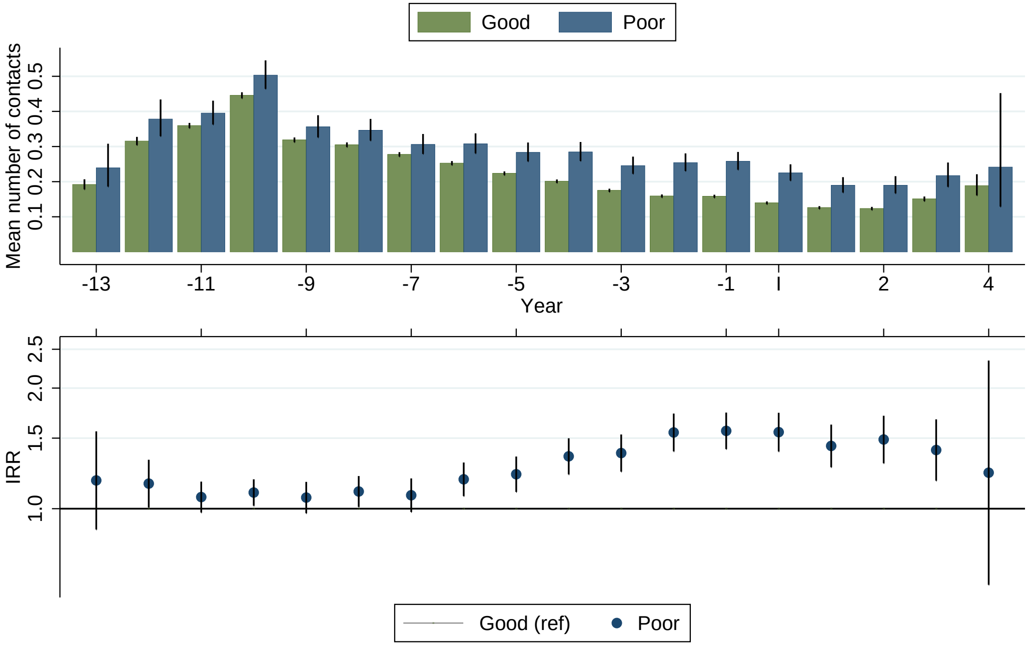

efig 4e. Spirometry

by overall self-rated health

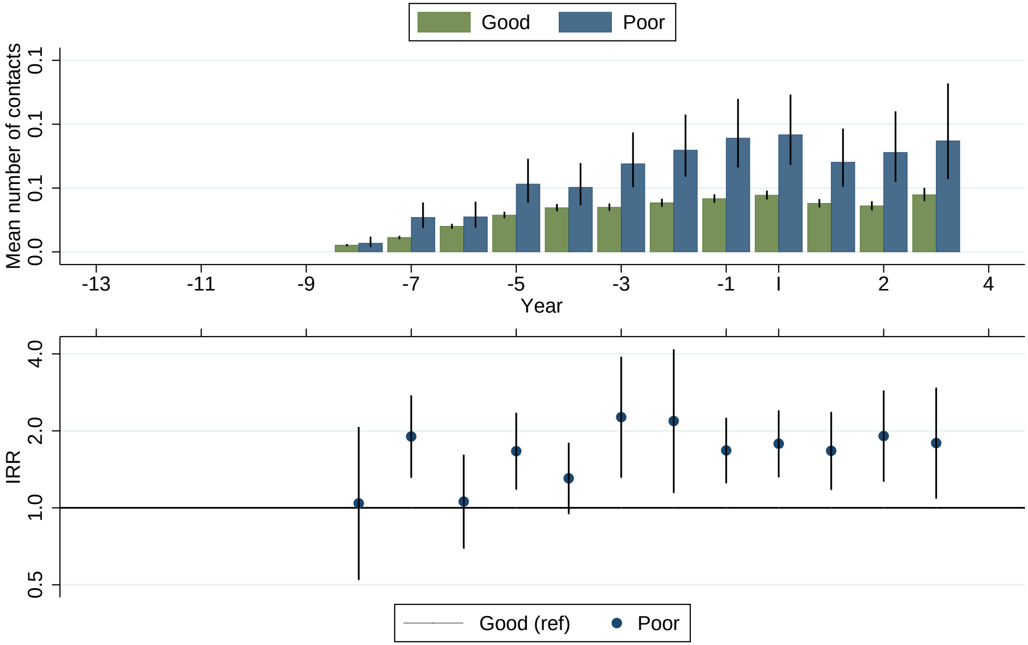

Supplement: Supplementary file 1 — Supplementary Information. [file 41598_2020_60125_MOESM1_ESM.pdf]
